# Data Management in a Regulatory Context

**DOI:** 10.3389/fmed.2017.00114

**Published:** 2017-07-21

**Authors:** Niels Grønning

**Affiliations:** ^1^SAS Institute, Copenhagen, Denmark

**Keywords:** ISO IDMP, SPOR, xEVMPD, European Medicines Agency, ICSR, Information Management, Data Management, Real-world evidence

## Abstract

With the implementation of Article 57(2) in 2012 the European Medicines Agency (EMA) embarked on a digitalization journey that foreseeably would ensure greater product oversight and interoperability across the community. This initiative has subsequently led to additional focus from the agency with respect to the utilization and harmonization of data as part of the regulatory process. Driven by both internal and external factors, the EMA have through the European Union telematics strategy laid the foundation for the regulatory-driven services that may be expected from the community the coming years. Supported by standardization initiatives (e.g., ISO Identification of Medicinal Products), the EMA is gradually building an information management-driven approach to data utilization and exploitation within drug evaluation and approval. Primarily driven by the increasing demand for signal detection, the EMA is additionally hoping to leverage the establishment of defined information models and supporting controlled terms to safeguard future activities within the community. Collectively, the overall community may seek to gain from the overall digitalization roadmap proposed by the EMA and interesting opportunities may be sought as part of the transition. Already now pharmaceutical companies are gradually adapting to this new paradigm and actively seeking to explore how they may leverage the future EMA operating model to serve internal business requirements. If successful, the collective efforts from industry and regulators may lead to an unprecedented product oversight and offer regulators the opportunity to proactively drive corrective actions and, therefore, improve patient safety.

## Introduction

For the pharmaceutical industry, the ability to successfully gather, analyze, compile, and submit regulatory information increasingly is perceived as a core operational capability trait that if adequately mastered serves to support not only clinical trial applications but also ultimately the marketing authorization submission. This process has historically been supported by a structured approach to information compilation in the form of the common technical document (CTD) and from a data perspective exemplified by the CDISC tabulation models [now also enforced by the Food and Drug Administration (FDA)]. Reference terminologies [e.g., Medical Dictionary for Regulatory Activities (MedDRA) and SNOMED CT] furthermore support the regulatory review process and ensure a uniform and unambiguous approach to term semantics.

The bulk of the information submitted is, however, still unstructured (non-machine readable) and this notion has with growing requirements for regulatory oversight proven to be a non-viable solution. In addition, requirements to mediate platform-independent interoperability between disparate industries (e.g., pharma and healthcare) increasingly drive the development of new cross regional reference terminologies and information models.

Since the discontinuation of the product information management (PIM) initiative in 2011 (1) additional ambitious initiatives have been set in motion within the European Union (EU) to increasingly focus on data management and the operational benefits pursued across the value chain. Driven by the European Medicines Agency (EMA), these initiatives seek to fundamentally alter the existing pharmaceutical operating model both from industry and agency perspective.

Faced with technical, political, and procedural complexity, the EMA has embarked on an interesting journey that if successful could fundamentally alter the drug approval process. This paper will address how the EMA is facilitating regulatory data management and interoperability [beyond article 57(2)] within the community and the prospects expected to be realized in the wake of medicinal and investigational product data standardization.

## Adopting Centralized Product Information

Introduced by Article 57(2) of Regulation [European Commission (EC)] No 726/2004 (2), the EMA has since 2 July 2012 enforced the industry facilitated submission and maintenance of medicinal product information. From an agency perspective, the initial focus was to build a complete repository of all human medicines for use in the European Economic Area (EEA) (3). The agency ambition was to utilize this information with respect to pharmacovigilance fee management, transparency, and to adequately support signal detection throughout the community. For the first time, pharmaceutical companies with products on the market in the EEA were requested to identify, standardize, submit, and manage regulatory data in an external agency facilitated data entry portal entitled EVWeb.

The industry-driven submission process was initially mediated by a largely manual-driven process [i.e., identify the adequate information in the respective summary of product characteristics and type the information into the dedicated data entry tool (EVWeb)]. The legal requirement brought with it new business processes related to the marketing authorization procedure (e.g., companies are required to submit the updated product information no later than 15 days following the reception of the marketing authorization approval) and, in some instances, a requirement for dedicated corporate roles to secure compliance with EMA requirements.

The manual-driven approach reflected the initial industry perception of the regulation as largely compliance driven rather than a regulation that facilitated not only agency but also industry benefits. Due to the general poor data quality post submission of the xEVMPD data, skepticism was initially raised by industry with respect to the subsequent utilization of the submitted information. To this day, the EMA is admittedly still struggling to adequately identify areas where the Article 57(2) regulation has significantly influenced the agency from an operational or cost perspective.

Ironically the implementation of Article 57(2) proved to be the first serious attempt (excluding the PIM initiative) from the EMA to pave the way for subsequent community initiatives leading to greater internal interoperability and data exchange between regulatory bodies and industry.

## What is Driving Change within the EMA?

Since the introduction of Article 57(2), the EMA has undergone significant internal changes to accommodate the incremental requirement for successful data exchange between industry, regulatory bodies, and the public. From an operational aspect, the agency is increasingly leveraging IT and information management to seek efficiency gains serving the general community and the pharmaceutical industry. This has led to the introduction of both regional initiatives (e.g., electronic application form and the PSUR repository) but also global initiatives [e.g., E2B(R2)].

Despite these initiatives, several factors still influence the EMA with regard to the reception, handling, and analysis of regulatory information. Two examples that drive change within the EMA are mentioned below representing external and internal factors, respectively.

## Eudravigilance and E2B(R3)

The submission of the electronic individual case safety reports (ICSR) to the EMA EudraVigilance database has historically served to safeguard the data foundation supporting adequate signal detection within the community. The data elements and technical requirements set forth in the E2B(R2) implementation guide furthermore ensures a consistent and uniform reporting mechanism to the EMA and in turn trust in the data analyzed.

However, growing requirements to integrate across the broader healthcare community has led to the notion that the current operating model and standardization formats may not be fully capable to support the future of cross-industry interoperability. In addition, the current inability to bridge ICSRs to discrete product entities (e.g., packaging, ingredients, batch numbers, etc.) has facilitated a demand to change the current E2B format and with that the generation of unique identifiers that span the breadth of the product information.

To facilitate the transition to E2B(R3), the EMA is required to enable the establishment of uniform coding to secure the adequate identification of product entities that potentially is influencing the benefit–risk profile of investigational and authorized products. On 22 May, the EMA management board officially approved the go-live of the next EudraVigilance release (scheduled for 22 November 2017) (4) that will support the new E2B(R3) format.

## The EMA Application Landscape

From an IT application perspective, the EMA have historically built a legacy IT architecture that supports a range of isolated business services across both industry and the respective member states. These business requirements have led to a silo-based application landscape [e.g., EudraGMDP, EudraCT, EudraPharm, European Review System, Article 57(2) database, etc.] dominated by cross application data duplication, disparate data, and system governance.

A general lack of data quality has significantly impaired the operational aspects within the agency and has further complicated the future requirements to conduct large-scale data analysis across the application landscape. The existing architecture and lack of data governance has proven inadequate to support the growing demand for data analytics.

Change is in turn mandated to secure the future architecture with respects to the overall digitalization aspirations within the community.

## How is Change Facilitated by the EMA?

With the first publication of the ambitious EU telematics strategy in 2014 (5) both the EMA and the heads of medicines agency collectively shared their large-scale plan for implementation of digitalization initiatives within the community. Subsequent updates following consultation with national competent authorities (NCA) and industry led to the current implementation roadmap covering 2015–2017 (6). For companies operating within the EU, it is highly advisable to consult this document since it offers detailed information potentially implicating not only internal processes but potentially also influencing internal data management principles and strategic technology choices. The strategy seeks to accommodate the continuous requirements for digitalization and transparency within the community and is in principle a prerequisite to accommodate legislative measures imposed by the EC.

## Master Data Management

It is evident from the thoughts and implementation schemas shared in the strategy that EMA is seeking to fundamentally alter their internal operating model for data management. Driven by the EMA Master Data Mangement (MDM) program entitled SPOR (Substance, Product, Organization and Referentials), pharmaceutical companies will be required to align to the future operating model and data standardization requirements set forth by the agency. Companies that perceived the implementation of Article 57(2) as a one off exercise will appreciate that this initial exercise was merely the first step toward greater data harmonization and transparency within the EU.

To fully appreciate the underlying foundation of the EMA MDM program, readers must understand that the EMA is utilizing the International Organization for Standardization (ISO) driven Identification of Medicinal Products (IDMP) information models (and implementation guidelines) currently being finalized. The ISO 11615 information model supports the structuring of medicinal and investigational medicinal product information across a large array of data domains names. Shared by the ISO 11615 model is the requirement to associate information structured in the ISO 11238 information model that spans both inactive and active substances. The information models support an extensive product granularity but also more importantly the ability to generate unique identifiers for pharmacovigilance purposes.

Based on the above-mentioned standards, the EMA will the coming years launch several IT services that collectively support the aspirations for greater regulatory data oversight, harmonization, and pharmacovigilance oversight across the community (7). The first applications (supporting the referentials and organizations) are expected to be launched within 2017 (8). Industry will be similar to the Article 57(2) requirements be required to submit and maintain information in these systems. Initially, the information required will be administrative (e.g., marketing authorization holder address, manufacturer address, and sponsor address) but within 2019 industry will be required to submit product and substance-related information to these IT systems (8).

The change facilitated by the agency allows for regulators and industry to successfully structure regulatory information per well-defined information models supported by centrally managed controlled terms. The ISO IDMP information models will in addition secure the availability of the unique identifiers required to accommodate the future E2B(R3) reporting requirements, thus, underlining the intricate relationship between the product information model in the ISO standards and the pharmacovigilance data requirements found on the E2B(R3) implementation guides.

The EU strategic direction is clearly mandating a more harmonized and standardized regulatory procedure forcing industry to adapt to the EMAs target operating model and supporting terms. Through an iterative process, the collective EMA business services (dominated by the SPOR MDM roadmap) will rely on information that is centrally managed and exposed to external parties (e.g., pharmaceutical companies, NCA and the public).

## Incremental EMA-Driven Initiatives to Drive Large Scale Opportunities

The EMA-driven digitalization journey that started with the Article 57(2) submission should not be perceived as failed attempt to structure regulatory information. Followed suite by the ambitious SPOR MDM roadmap, it is evident that the agency the coming years will lead the structured data collection and management of regulatory information across the community.

This regulatory information foundation will serve as the basis supporting additional agency-driven initiatives (e.g., falsified medicines directive, clinical trial portal, etc.) and is in turn critical to adequately drive future IT initiatives within the community. The impetus to act should in turn be seen from a broader perspective where the EMA is seeking to explore the foundational aspects of information management and some of the opportunities this may drive.

In acknowledgment that the foundation for ISO IDMP is to improve the overall signal detection within the community the EMA may build internal capabilities to continuously monitor and impose corrective actions (e.g., batch recalls, labeling updates, etc.) based on near real-time data submitted to the agency. These activities may ultimately not only improve the overall operating model within the agency but, more importantly, also support greater patient safety across the community.

With SPOR, the long-term possibility to support regulatory data exchange in the form of structured product labeling (SPL) rather than CTD leaves some interesting aspects of the future operating model within the EMA. Already, we have seen examples of this transition exemplified by possibility to submit minor variations (e.g., location of the PSMF and Qualified Person Pharmacovigilance updates) (9) through the xEVMPD database rather than the existing document-driven process.

This transition may very well prove beneficiary not only from the agency point of view but also from industry since it addresses the requirements to adequately react to product changes and notify the regulators through a less ridged procedure.

From a financial perspective, the increasing digitalization of regulatory processes may prove to refocus the investments to research and development as compared to the costly lifecycle management process. The opportunities EMA may seek as part of the SPOR initiative safeguards not only regulatory oversight but also drives the transition toward an increasingly proactive role as part of the drug development and evaluation process.

## Leveraging Regulatory-Driven Compliance While Harvesting Corporate Benefits

From an industry perspective, the implementation of SPOR in the EU sets forth some interesting perspectives for the implementation of cross organizational data management principles. Hindered by existing legacy IT systems, silo-based applications and fragmented data management strategies companies may gradually start accepting the possibilities SPOR and in particular ISO IDMP may offer. In addition, companies may adopt the regulatory-driven data definitions and semantics across the enterprise to facilitate the establishment of internal data governance principles.

The EMA requirement to submit ISO IDMP information based on the SPL document markup standard additionally leaves for some interesting perspectives with respect to the entire CTD generation process. From a regulatory business process perspective, companies may with time implement capabilities supporting the generation of regulatory documentation (e.g., CTD) based on the Extensible Markup Language (xml) format from adequate controlled IT sources. This procedural change may support greater harmonization with respect to the documentation submitted globally as well as enhanced capabilities with relation to future variation submissions and labeling updates.

## Transition to Digital Regulatory Information Management

Considering the ambitious digitalization plans initiated by the EMA industry may experience that the agency will strive to continuously mandate the harmonized data transmission between community stakeholders and the EMA through unified exchange standards [e.g., SPL and E2B(R3)]. From a global perspective, the implementation of global (and not regional) standards adequately paves the way for greater interoperability between regions (e.g., US and EU). Already the most recent Prescription Drug User Fee Act (PDUFA) (10) includes a clear intent to utilize the ISO IDMP standards with respect to the future regulatory business processes in the United States (US). Both the PDUFA and EU telematics strategy are expected to be updated within 2017 and it will be interesting to follow the future strategic direction exhibited by both regulatory bodies with respect to data standardization initiatives. Clearly, the legal constraints associated with data sharing across the regions (e.g., US and EU) may significantly impede the ability to successfully exploit data insight and we may find that data analysis may be conducted from a purely regional perspective.

Exploring the agency-driven digital aspirations beyond data standardization, the EMA is also increasingly seeking to promote data transparency. It is the agencies extent to utilize the information from the Article 57(2) database [currently holding 673,134 medicinal products (11)] as part of the future EU Medicines Web Portal (replacing EudraPharm) (12). We may very well see additional externalization of data as part of future agency-driven initiatives. This will increasingly mandate that industry maintains data quality to adequately secure the correct presentation of product information in the public space.

Collectively, the EMA and industry have embarked on an interesting journey. With the activities mentioned in this paper, the entire submission and lifecycle management process may undergo significant revisions the coming years. Expectations to supplement CTD documents with data per established data models and terminologies leave the opportunity to gradually decommission the document-driven process and solely rely on data for review purposes. This fundamentally alters the operating model within industry and health authorities but also lays the foundation for data utilization and interoperability across industry segments (e.g., Pharmaceutics and Healthcare). From a payor, industry and regulatory perspective the exploitation of real-world data (e.g., from patient registries) demands harmonized standards, exchange schemas and information models to secure uniform data analytics and exchange between the respective stakeholders. This foundation will strengthen the transition towards cross-industry interoperabiity as well as the incremental implementation of a more data-centric drug approval process.

## Author Contributions

The author confirms being the sole contributor of this work and approved it for publication.

## Conflict of Interest Statement

The author declares that the research was conducted in the absence of any commercial or financial relationships that could be construed as a potential conflict of interest.
